# Catalytic Synergy: Mesoporous Silica and Ruthenium—Structure–Activity Relationships in CO_2_ Methanation and Toluene Hydrogenation

**DOI:** 10.3390/molecules31071130

**Published:** 2026-03-29

**Authors:** Ewa Janiszewska, Mariusz Pietrowski, Michał Zieliński

**Affiliations:** Faculty of Chemistry, Adam Mickiewicz University, Uniwersytetu Poznańskiego 8, 61-614 Poznań, Poland; eszym@amu.edu.pl (E.J.); mariop@amu.edu.pl (M.P.)

**Keywords:** ruthenium catalyst, SBA supports, toluene hydrogenation, CO_2_ hydrogenation

## Abstract

The rational design of supported ruthenium catalysts for sustainable energy applications requires precise control over metal nanoparticle size, dispersion, and metal–support interactions. This study investigates the influence of mesoporous silica support topology—SBA-15 (2D hexagonal, cylindrical pores), SBA-12 (3D hexagonal structure), and SBA-3 (2D hexagonal)—on the structure and catalytic performance of 1 wt% ruthenium catalysts in CO_2_ methanation and gas-phase toluene hydrogenation. Comprehensive characterization by nitrogen physisorption, low- and high-angle X-ray diffraction (XRD), H_2_ temperature-programmed reduction (H_2_-TPR), CO chemisorption, and transmission electron microscopy (TEM) revealed that support pore architecture dictates ruthenium particle size (1.2 nm for Ru/SBA-15, 2.8 nm for Ru/SBA-3, 4.3 nm for Ru/SBA-12) and dispersion (80%, 35%, 23%, respectively) through geometric confinement effects. Catalytic testing demonstrated contrasting structure–activity relationships: CO_2_ methanation exhibited strong structure sensitivity with turnover frequency (TOF) increasing with particle size (Pearson’s r = 0.96), favoring Ru/SBA-3 and Ru/SBA-12 with near-optimal 3–4 nm particles, while toluene hydrogenation showed weaker structure sensitivity, with Ru/SBA-12 achieving the highest TOF owing to its larger particle size and higher crystallinity. These findings underscore the critical importance of tailoring mesoporous support topology to match reaction-specific structure sensitivity, providing fundamental insights for the design of bifunctional catalysts for hydrogenation reactions.

## 1. Introduction

The transition toward sustainable chemical manufacturing and reduced carbon emissions has driven the search for highly efficient catalytic systems capable of operating under mild conditions [1,2,3,4]. Hydrogenation reactions represent one of the most important classes of catalytic transformations in the chemical and petrochemical industries [5,6,7]. They are used extensively for aromatic ring saturation, olefin reduction, and hydrodeoxygenation processes, enabling the production of fuels, lubricants, and polymer intermediates [8,9,10]. Conventional hydrogenation processes typically require high temperatures and pressures; however, the development of catalysts that maintain high activity and selectivity at lower temperatures is essential for energy-efficient and environmentally benign operations [6,11,12,13]. In this context, ruthenium-based catalysts supported on mesoporous materials have attracted considerable attention due to their high intrinsic activity in hydrogenation reactions, tunable metal–support interactions, and enhanced dispersion within ordered mesoporous pore networks [13,14,15,16,17,18]. Despite their promising catalytic potential, the performance of ruthenium catalysts is strongly influenced by the physicochemical characteristics of the support. The appropriate choice of support not only affects metal dispersion and particle size but also determines the strength of metal–support interactions and the accessibility of active sites, which collectively govern catalytic performance.

Ordered mesoporous silica materials have emerged as versatile catalyst supports owing to their high surface areas, uniform pore systems, and structural tunability, which enable precise control over metal dispersion and accessibility of active sites. Early representatives of this class, such as MCM-type materials (e.g., MCM-41 and MCM-48), exhibit ordered mesoporous architectures but often suffer from limited hydrothermal stability and relatively thin pore walls, which may lead to structural degradation under demanding catalytic conditions [19,20]. In contrast, the Santa Barbara Amorphous (SBA) family of mesoporous silicas offers enhanced thermal and hydrothermal stability, thicker silica walls, and improved structural robustness, making these materials particularly attractive for catalytic applications [21,22,23].

In recent years, ordered mesoporous silica materials such as SBA-3, SBA-12, and SBA-15 have gained considerable attention as catalyst supports. Their well-defined pore structures, large surface areas, tunable pore diameters, and chemical inertness make them ideal platforms for the dispersion and confinement of metallic nanoparticles [24,25,26,27,28]. SBA-3 is characterized by a two-dimensional hexagonal array of narrow, cylindrical mesopores with diameters of approximately 2 nm, providing strong spatial confinement for supported metal species [25,26]. SBA-12 possesses a three-dimensionally interconnected hexagonal mesostructure composed of cage-type pores with typical diameters of 3–4 nm, which enables multidirectional mass transport while retaining a confinement effect [27,28]. In contrast, SBA-15 exhibits larger and uniform one-dimensional channels (5–6 nm) along with high hydrothermal stability resulting from its thick silica walls, facilitating enhanced diffusion of reactant molecules and greater tolerance toward metal particle growth [24].

These pronounced textural and topological differences within the SBA family allow for a systematic investigation of structure–activity relationships in supported ruthenium catalysts, particularly with respect to metal dispersion, particle stability, and transport limitations in hydrogenation reactions carried out under mild conditions [29,30,31,32,33].

Therefore, the aim of this work is to systematically elucidate how the topology and pore architecture of ordered mesoporous silica supports from the SBA family influence the structural properties, metal dispersion, and catalytic behavior of supported ruthenium nanoparticles. By employing SBA-15, SBA-12, and SBA-3 as model supports with distinct pore dimensionality, size, and connectivity, this study seeks to isolate the effects of geometric confinement and metal–support interactions on ruthenium particle size and crystallinity. Furthermore, the catalytic consequences of these structural features are evaluated in two hydrogenation reactions with differing degrees of structure sensitivity, CO_2_ methanation and gas-phase toluene hydrogenation, allowing for a direct comparison of reaction-specific structure–activity relationships. Through this approach, the work aims to provide fundamental insights into rational support design strategies for optimizing ruthenium-based catalysts tailored to sustainable hydrogenation processes.

## 2. Results and Discussion

### 2.1. Nitrogen Adsorption-Desorption Analysis and Textural Properties (BET, BJH)

**Figure 1:**
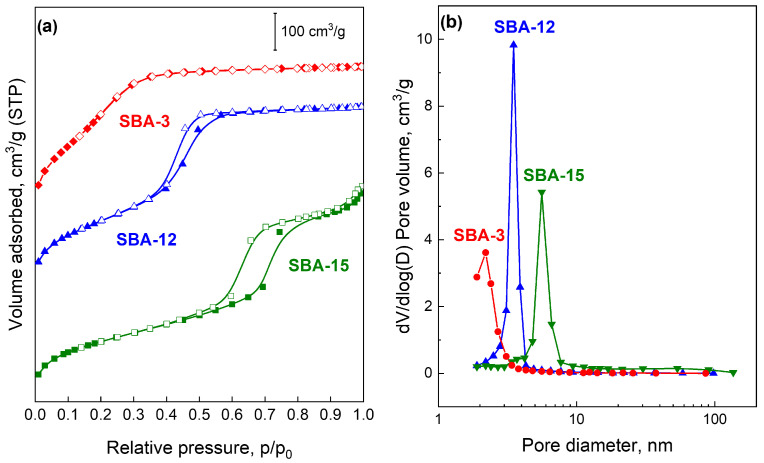
Nitrogen adsorption–desorption isotherms (the lines have been shifted along the Y axis to increase clarity): (**a**) and pore volume distribution as a function of pore size (**b**) of SBA-3, SBA-12 and SBA-15 supports calcined at 550 °C.

The nitrogen adsorption–desorption isotherms recorded at −196 °C (Figure 1a) show that all three materials exhibit type IV isotherms according to IUPAC classification [34], characteristic of well-defined mesoporous structures. Distinct hysteresis loops at p/p_0_ = 0.4–0.8 for SBA-12 and SBA-15 confirm capillary condensation in mesopores [35], whereas SBA-3 shows no hysteresis, consistent with literature data [36,37,38,39]. In SBA-3, capillary condensation is evidenced by a sharp step at p/p_0_ = 0.15–0.30, indicating smaller mesopores.

The SBA-15 support displays a type IVa isotherm with an H1 hysteresis loop (Figure 1a), typical of uniform cylindrical pores with narrow size distribution [40]. The condensation step at p/p_0_ ≈ 0.55–0.8 reflects high structural ordering and larger mesopores compared to SBA-3 and SBA-12 [41]. The isotherm shape corresponds to a 2D hexagonal (p6mm) structure commonly reported for SBA-15 [40].

The SBA-12 material also exhibits a type IVa isotherm, with a narrower hysteresis loop at slightly lower relative pressures (p/p_0_ ≈ 0.4–0.6). Its nearly vertical adsorption–desorption branches indicate a homogeneous pore network consistent with a 3D hexagonal structure (P6_3_/mmc symmetry) described in the literature [42].

SBA-3 presents a sharp nitrogen uptake at p/p_0_ = 0.15–0.30 without hysteresis, suggesting uniform, highly regular mesopores and rapid reversible condensation. The lower condensation pressure compared to SBA-12 and SBA-15 confirms that SBA-3 possesses the smallest mesopores [43]. Its isotherm shape indicates a 2D hexagonal structure similar to SBA-15 but with narrower channels [44].

BJH pore size distributions (Figure 1b) are narrow and monomodal for all samples, confirming high structural order. SBA-15 shows a sharp peak at 5–6 nm, typical for materials synthesized with Pluronic P123 [35]. A small micropore contribution (Table 1), associated with template penetration into silica walls, appears at low pore diameters. SBA-12 exhibits a maximum at ~4 nm, and the distribution shape reflects the dual-pore 3D hexagonal structure, with large spherical cavities and smaller connecting windows. BJH analysis (based on the Kelvin equation for cylindrical pores) may underestimate the size of spherical cavities in cage-like structures [45,46]. However, the distribution confirms interconnected mesopores in the 3D framework, beneficial for mass transport in catalytic applications. SBA-3 shows the narrowest distribution centered at ~2 nm, indicating highly uniform small mesopores suitable for stabilizing small metal nanoparticles and enhancing confinement effects [44].

BET surface areas decrease in the order: SBA-3 (1032 m^2^/g) > SBA-12 (845 m^2^/g) > SBA-15 (754 m^2^/g) (Table 1), consistent with literature reports [47]. The highest value for SBA-3 results from its small pore diameter and thin walls, maximizing the surface-to-volume ratio [48]. SBA-12 shows an intermediate surface area due to its 3D cage-like geometry [42], while SBA-15, despite having the largest pores (5–8 nm), exhibits the lowest surface area, reflecting the well-known inverse relationship between pore size and surface area in mesoporous silicas [49]. Nevertheless, its high structural order and large pores make SBA-15 particularly suitable for applications involving bulky molecules and catalyst supports [50].

The textural properties correlate with the structure of each mesoporous silica. SBA-15 and SBA-3 possess a 2D hexagonal (p6mm) structure with uniform, non-interconnected cylindrical pores, giving sharp pore size distributions and H1 hysteresis; their pore size difference (4–8 nm for SBA-15 vs. 2–4 nm for SBA-3) results from different synthesis conditions and templates [35,44]. In contrast, SBA-12 has a 3D hexagonal (P6_3_/mmc) cage–window structure with interconnected pores, offering improved molecular accessibility and diffusion, though with slightly lower surface area than the 2D materials.

The low-temperature nitrogen adsorption/desorption isotherms were used to determine the surface area, average pore diameter, and average pore volume of the 1 wt% ruthenium catalysts (Table 1 and Figure S1). Impregnation with the ruthenium precursor leads to a decrease in surface area and pore volume, accompanied by an increase in average pore diameter. These changes can be attributed to the partial filling of the mesopores by the ruthenium phase, resulting in a reduction in the diameter of medium and large pores and the blocking of the smallest pores. The smallest decrease in surface area (~4%) was observed for Ru/SBA-3, whereas the largest (~7%) occurred for Ru/SBA-12. The N_2_ isotherms and pore size distributions (Figure S1a,b) of the catalysts remain similar to those of the corresponding supports (Figure 1).

### 2.2. X-Ray Diffraction (XRD) Analysis of Mesoporous Supports and Ruthenium Catalysts

**Figure 2:**
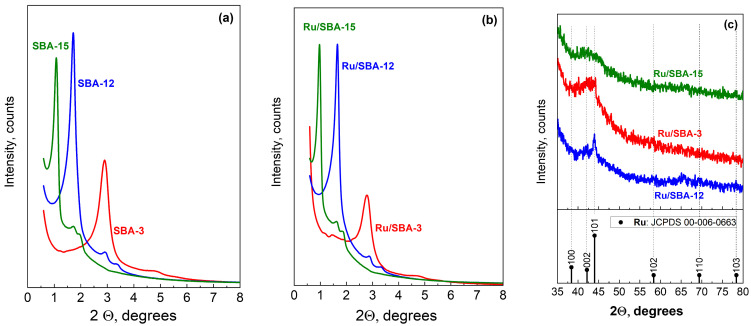
Low—(**a**,**b**) and wide—(**c**) angle XRD patterns of supports and catalysts.

The low-angle XRD patterns (2θ = 0.6–8°) confirm well-ordered mesostructures of all supports (Figure 2a). SBA-15 shows a sharp (100) reflection at ~1.5°, typical of a 2D hexagonal (p6mm) structure with high long-range order. SBA-12 exhibits a (002) peak at ~2° with additional reflections at 2.9° and 3.3° ((112) and (300)), consistent with its 3D hexagonal (P6_3_/mmc) network. SBA-3 displays the main reflection at ~3°, indicating the smallest unit cell and a 2D hexagonal structure. These patterns agree with literature data for ordered SBA materials [51].

After 1 wt% Ru impregnation and reduction at 450 °C, the characteristic low-angle peaks are largely preserved (Figure 2b), confirming retention of mesostructure. A slight intensity decrease is observed for Ru/SBA-3, suggesting partial loss of ordering, likely due to its lower hydrothermal stability. Ru/SBA-15 and Ru/SBA-12 maintain their main reflections with minor intensity changes, indicating that impregnation, drying (100 °C), and H_2_ reduction do not significantly affect mesoporosity, which is essential for accessible active sites and efficient mass transport.

High-angle XRD (2θ = 35–80°) shows no metallic Ru reflections for Ru/SBA-15 and Ru/SBA-3 (Figure 2c), indicating highly dispersed nanoparticles (~1–3 nm), below XRD detection limits. This agrees with CO chemisorption results showing dispersions of 80% (1.2 nm) for Ru/SBA-15 and 35% (2.8 nm) for Ru/SBA-3 (Table 2). Such absence of Ru^0^ peaks is typical for low metal loadings (≤1–3 wt%) on mesoporous silica, where strong metal–support interactions help stabilize small particles.

Only Ru/SBA-12 shows a weak peak at ~44° corresponding to the (101) reflection of hexagonal Ru (JCPDS 06-0663), indicating the presence of larger crystallites (>3–4 nm). This matches its lowest dispersion (23%) and largest particle size (4.3 nm) (Table 2). The formation of larger Ru particles in SBA-12 can be attributed to its 3D cage-like structure, which favors aggregation compared to the 1D cylindrical channels of SBA-15 and SBA-3, highlighting the key role of pore architecture in controlling metal dispersion.

### 2.3. Temperature-Programmed Reduction (H_2_-TPR) Analysis

**Figure 3:**
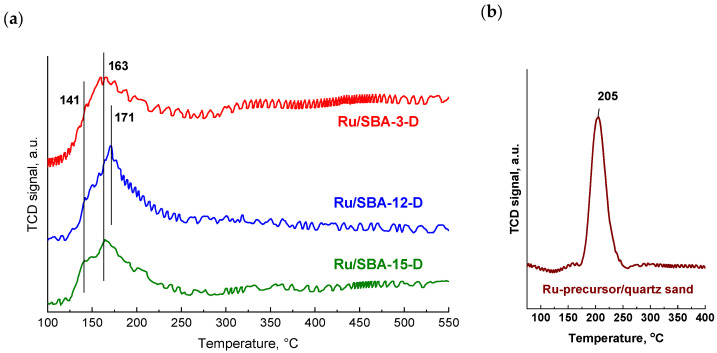
H_2_-TPR profiles of the dried Ru catalysts (**a**) and the reference RuCl_3_·nH_2_O/quartz sand sample (**b**). For comparison purposes, the signal intensities were normalized to a catalyst mass of 100 mg.

Figure 3 shows the H_2_-TPR patterns obtained for ruthenium catalysts supported on different mesoporous silica materials, highlighting variations in their reduction characteristics that arise from differences in ruthenium dispersion and metal–support interactions. All samples were prepared by incipient wetness impregnation with hydrated RuCl_3_ as the metal precursor and subsequently dried at 100 °C without a calcination step. As a result, ruthenium was present before reduction predominantly in the form of hydroxychloride species [Ru(OH)_x_Cl_y_] and/or hydrated ruthenium oxides. In contrast, the corresponding silica supports exhibited no reduction features in their H_2_-TPR profiles, confirming their non-reducible nature.

A preliminary examination of the H_2_-TPR profiles indicates that the temperatures at which the maximum reduction of the ruthenium precursor occurs vary depending on the type of support used. Such differences point to variations in precursor–support interaction strength and/or in the dispersion of the ruthenium species on the support surface. In the H_2_-TPR analysis, quartz sand impregnated with RuCl_3_·nH_2_O was employed as a reference material (Figure 3b). For this Ru precursor deposited on quartz, the reduction proceeded in a single step, with a peak centered at approximately 205 °C. Similar single-step reduction of RuCl_3_·nH_2_O directly to metallic ruthenium has been reported in the literature [52], where it was also demonstrated that such a procedure, without a calcination step, promotes the formation of finely dispersed Ru crystallites. Compared to the Ru/quartz reference, catalysts supported on mesoporous silica exhibited reduction features shifted toward lower temperatures, which can be attributed to enhanced dispersion of the active phase. As reported by Mazzieri et al. [53], catalysts derived from RuCl_3_ typically display reduction signals in the 140–220 °C range, associated with the sequential reduction of RuO_2_ or Ru(OH)Cl_3_ species.

The H_2_-TPR profiles of the three ruthenium catalysts reveal distinct reduction behaviors that correlate strongly with their support pore architectures and the resulting metal–support interactions. The total degree of reduction of the supported ruthenium precursor, calculated based on hydrogen consumption in the temperature range RT-550 °C, exceeded 95% for Ru/SBA-3 (95.2%), Ru/SBA-12 (97.7%) and Ru/SBA-15 (98.5%) catalysts, indicating an almost complete reduction of the active phase and the presence of ruthenium predominantly in the catalyst at zero oxidation state.

Ru/SBA-12 exhibits the highest reduction temperature with a maximum at 171 °C, displaying a relatively sharp and symmetrical peak that reflects the presence of Ru species with intermediate interaction strength with the 3D hexagonal support structure; this elevated reduction temperature, combined with the appearance of the (101) Ru^0^ reflection in high-angle XRD—Figure 2c, suggests that the cage-like pore topology of SBA-12 facilitates the formation of somewhat larger and more aggregated Ru particles within the interconnected cavities, requiring higher thermal energy for complete reduction of Ru_x_O_y_Cl species to metallic Ru^0^.

Ru/SBA-3 and Ru/SBA-15 both show reduction maxima at 163 °C, indicating their broadly similar reduction kinetics, yet their peak shapes differ significantly: Ru/SBA-3 presents a relatively narrow and intense peak consistent with a more uniform population of Ru species predominantly located on the external surface or near pore openings due to restricted access to the smaller pores (2–3 nm), while Ru/SBA-15 displays a broader reduction profile with a pronounced shoulder at 141 °C that extends to higher temperatures, reflecting a heterogeneous distribution of Ru species with varying interaction strengths—the low-temperature shoulder at 141 °C likely corresponds to weakly interacting or more exposed Ru species near pore mouths, whereas the main reduction event centered at 163 °C represents Ru nanoparticles deeply confined within the large cylindrical mesopores (5–7 nm), where strong Ru-O-Si anchoring and geometric confinement effects stabilize highly dispersed ultra-small particles (1.2 nm, 80% dispersion—Table 2) that require sustained hydrogen exposure for complete reduction. This interpretation aligns with the literature, which demonstrates that the metal–support interaction strength inversely correlates with the reduction temperature for weakly interacting supports, such as silica. Additionally, confinement effects in ordered mesoporous materials can create heterogeneous reduction environments, depending on the particle’s location within the pore network [54,55]. The bimodal character of Ru/SBA-15’s H_2_-TPR profile, observed to a lesser extent for Ru/SBA-3 and Ru/SBA-12, provides compelling evidence that the large, uniform cylindrical channels of SBA-15 uniquely enable deep penetration and stabilization of ultra-small Ru nanoparticles throughout the entire pore volume, creating a gradient of metal–support interaction strengths from pore openings to the interior that manifests as the characteristic broad, asymmetric reduction profile with a distinct low-temperature shoulder.

### 2.4. CO Chemisorption Analysis

The CO chemisorption results, presented in Table 2, provide crucial information regarding ruthenium dispersion and metallic particle size in the investigated catalysts, which can be correlated with the H_2_-TPR profiles discussed previously. The observed relationship between reduction temperature and the textural properties of ruthenium species offers valuable insights into the influence of support structure on the state and accessibility of the active phase. For comparison of catalytic trends, CO chemisorption measurements were also performed for a Ru catalyst supported on commercial non-ordered SiO_2_ (Table S1), providing additional information on catalysts with larger Ru particle sizes than those observed for the SBA-based materials.

Ru/SBA-15 presents an apparent paradox: despite showing the lowest onset reduction temperature (shoulder at 141 °C) and a broad TPR profile extending to 163 °C—Figure 3a—it achieves the highest dispersion (80%) and smallest particle size (1.2 nm)—both confirmed by CO chemisorption (Table 2) and the absence of XRD-visible Ru crystallites—Figure 2c. This counterintuitive behavior can be rationalized by considering that the TPR profile reflects the reduction kinetics of Ru precursor species (RuCl_3_·xH_2_O → RuO_x_Clᵧ → Ru^0^), not the final metal particle size. The bimodal TPR character suggests two distinct species: (1) easily reducible Ru species near pore openings or external surfaces (141 °C shoulder), contributing to the moderate dispersion component, and (2) Ru species deeply anchored within the large cylindrical mesopores (5–7 nm) through strong Ru-O-Si interactions (163 °C peak), which, paradoxically, yield the most highly dispersed Ru nanoparticles due to geometric confinement and spatial isolation effects within the uniform 1D channels. Literature demonstrates that metal nanoparticles confined in cylindrical mesopores experience restricted mobility and coalescence during reduction, with the confinement effect becoming stronger as the particle-to-pore size ratio decreases [56]; thus, SBA-15’s large pores (5–7 nm) can accommodate and stabilize ultra-small Ru clusters (1.2 nm) throughout the entire pore volume, preventing sintering even after complete reduction.

Ru/SBA-3 displays an intermediate reduction temperature (163 °C—Figure 3a) with a relatively narrow, intense peak, yielding moderate dispersion (35%) and intermediate particle size (2.8 nm), with no XRD-visible Ru crystallites. The H_2_-TPR profile indicates relatively uniform Ru speciation, likely dominated by surface or near-surface species, due to the smaller pore dimensions (2–3 nm) that restrict deep precursor penetration during impregnation. The correlation between H_2_-TPR and dispersion here follows classical behavior: the moderate reduction temperature reflects moderate metal–support interaction, and the narrow peak shape indicates a homogeneous population of Ru species, predominantly located on the external surface or within the first few nanometers of pore openings. The absence of XRD reflections confirms that particles remain below 3 nm, consistent with the 2.8 nm average from chemisorption. Compared to SBA-15, the lack of a clearly defined low-temperature shoulder in Ru/SBA-3’s TPR profile suggests a more uniform interaction environment, likely because the small pores (2–3 nm) provide limited confinement space—Ru particles cannot penetrate deeply, so there is no gradient of interaction strengths from external to internal sites.

Ru/SBA-12 exhibits the highest reduction temperature (171 °C) with a sharp, symmetrical peak, yet shows moderate-to-low dispersion (23%) and the largest particle size (4.3 nm), with XRD-visible Ru^0^ crystallites evidenced by the (101) reflection at 44°. This correlation is more intuitive: the elevated reduction temperature indicates that Ru precursors within the 3D hexagonal structure (~3–4 nm windows connecting larger cavities) experience hindered hydrogen diffusion and restricted access, requiring higher temperatures to achieve complete reduction. However, the pore topology of SBA-12 inherently promotes particle aggregation—once Ru_3_Cl_x_ species are reduced to mobile Ru^0^ atoms at 171 °C, they can migrate within the interconnected cage network and coalesce into larger crystallites (4.3 nm) that approach or exceed the cage dimensions, as confirmed by XRD. Literature on metal confinement in cage-type mesoporous materials (KIT-6, SBA-16) shows that 3D pore networks, while beneficial for mass transport, provide less effective spatial isolation than 1D cylindrical channels, allowing metal atoms to diffuse between adjacent cages and form larger aggregates [57]. The sharp TPR peak also suggests a more homogeneous reduction environment compared to SBA-15, consistent with Ru species being predominantly located within similar cage environments rather than distributed along a gradient from pore mouths to channel interiors.

### 2.5. Transmission Electron Microscopy Analysis

The changes in mean Ru particle size were characterized by TEM imaging. Transmission electron microscopy revealed clear differences in Ru dispersion depending on the type of mesoporous silica support—Figure 3.

**Figure 4:**
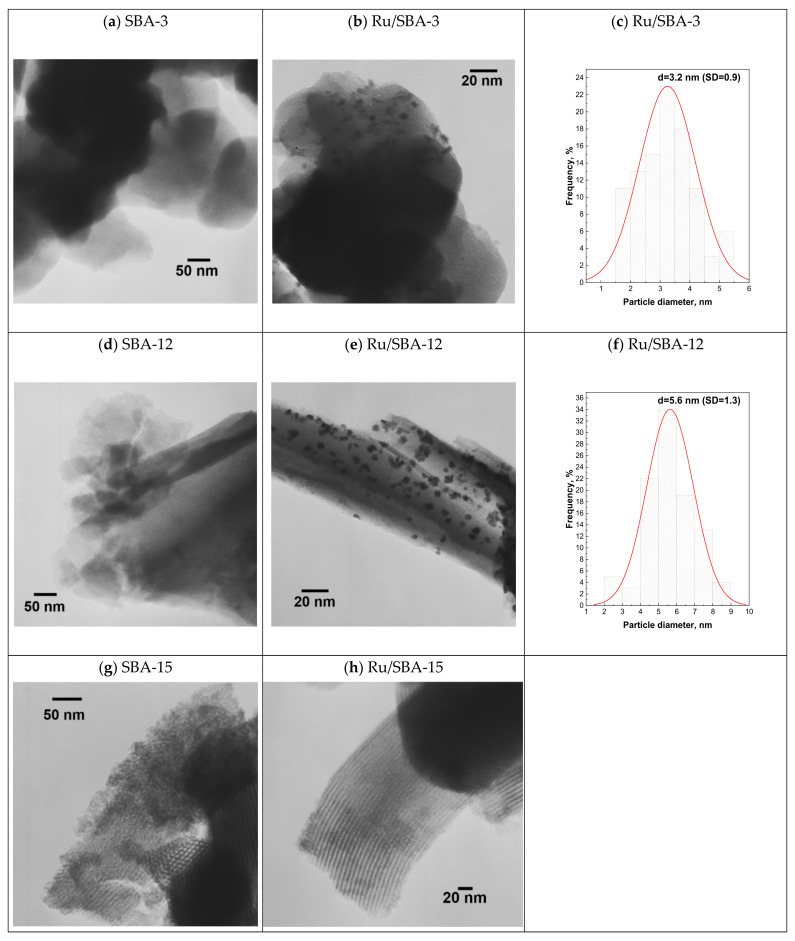
TEM micrographs of the SBA support (**a**,**d**,**g**), Ru/SBA catalysts (**b**,**e**,**h**), and particle size distribution of the Ru/SBA catalysts (**c**,**f**).

For Ru supported on SBA-3, well-dispersed Ru nanoparticles were observed with a relatively narrow particle size distribution (Figure 4a–c). The mean particle size determined from TEM analysis was 3.2 ± 0.9 nm. This value is in very good agreement with the mean particle size calculated from CO chemisorption (2.8 nm—Table 2). The slight difference between the two methods (ca. 14%) falls within the statistical uncertainty of the TEM measurement and confirms consistent estimation of Ru dispersion. The good agreement indicates uniform nanoparticle distribution and negligible aggregation in this system.

In contrast, Ru supported on SBA-12 exhibited a broader particle size distribution (5.6 ± 1.3 nm—Figure 4d–f) and the presence of partially aggregated Ru nanoparticles—Figure S2. The mean particle size derived from CO chemisorption (4.3 nm) was smaller than that obtained from TEM. This discrepancy can be attributed to the formation of aggregates, which increase the number-averaged particle size in TEM analysis, while CO chemisorption reflects only surface-accessible Ru atoms0. Ru atoms located within aggregates are not fully accessible for CO adsorption, leading to a smaller effective particle size calculated from chemisorption data. The larger standard deviation observed for Ru/SBA-12 further confirms the higher heterogeneity of metal dispersion on this support.

A distinctly different behavior was observed for Ru supported on SBA-15—Figure 4g,h. No clearly distinguishable Ru nanoparticles were detected in conventional TEM images (Figure 4h). However, CO chemisorption measurements indicate a mean particle size of 1.2 nm, corresponding to highly dispersed ultrasmall Ru clusters. The absence of visible particles in TEM is therefore attributed to the subnanometric size of the Ru species, which approaches the detection limit of conventional bright-field TEM due to low contrast against the silica framework. Moreover, the lack of observable Ru on TEM images may also suggest that the Ru clusters are located inside the pores of SBA-15, which has the largest pores among the tested supports (compared to SBA-3 and SBA-12). The well-ordered hexagonal pore structure and thicker pore walls of SBA-15 likely promote uniform nucleation and effectively suppress Ru nanoparticle aggregation, resulting in exceptionally high metal dispersion.

### 2.6. Catalytic Tests—Hydrogenation of Toluene and Selective Hydrogenation of CO_2_ to Methane

**Figure 5:**
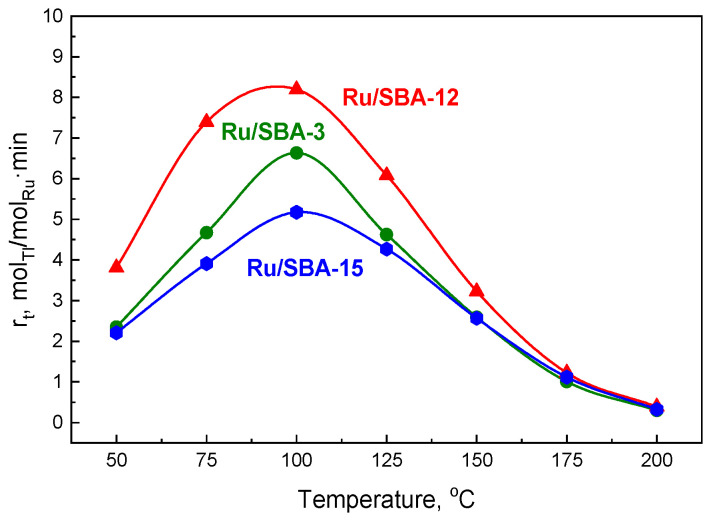
Influence of the catalyst support on the apparent toluene hydrogenation rate, normalized to the total ruthenium content, as a function of temperature. Catalyst pre-treatment was carried out at 450 °C under a hydrogen flow of 100 cm^3^/min for 2 h. Hydrogenation experiments were performed with a toluene concentration of 0.75 µmol/cm^3^ and a hydrogen flow rate of 50 cm^3^/min; catalytic activity was evaluated after 20 min on stream.

**Figure 6:**
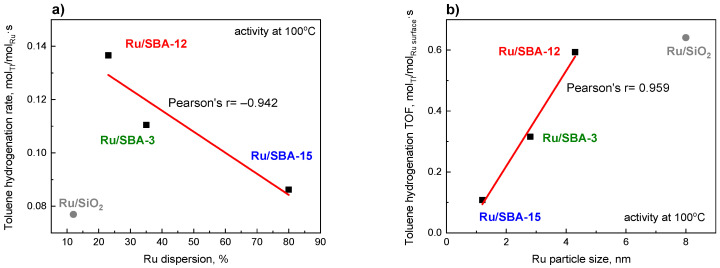
Toluene hydrogenation: rate as a function of Ru dispersion (**a**) and TOF as a function of Ru particle size (**b**). The Ru/SiO_2_ data point was not included in the linear fit. Activation of catalyst: 450 °C, H_2_ = 100 cm^3^/min, 2 h; Reaction: C_Tl_ = 0.75 μmol/cm^3^, H_2_ = 50 cm^3^/min, activity after 20 min, T_reac_ = 100 °C.

**Figure 7:**
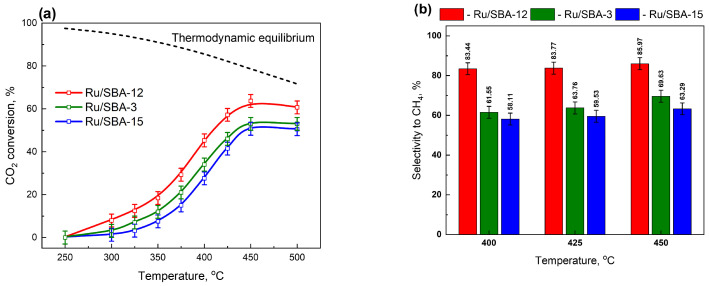
Catalytic performance of Ru catalysts: CO_2_ conversion (**a**) and CH_4_ selectivity (**b**). Prior to the reaction, all samples (50 mg) were reduced under H_2_ at 450 °C for 2 h. The reactant gas mixture consisted of CO_2_:H_2_:He = 1:4:5, with a total flow rate of 100 cm^3^/min.

**Figure 8:**
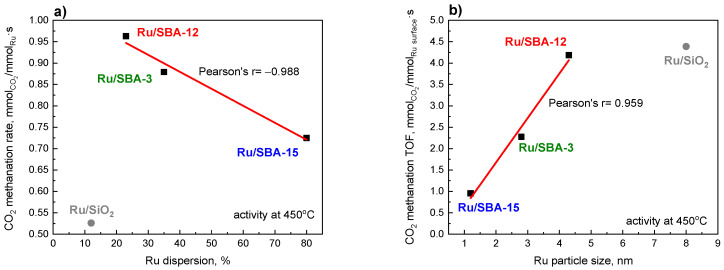
CO_2_ methanation: rate as a function of Ru dispersion (**a**) and TOF as a function of Ru particle size (**b**). The Ru/SiO_2_ data point was not included in the linear fit. Reaction conditions: before reaction all catalysts (50 mg) were reduced in H_2_ at 450 °C, 2h; the feed gas composition—CO_2_:H_2_:He = 1:4:5; total flow rate—100 cm^3^/min; T_reac_ = 450 °C.

The catalytic activity of the obtained catalysts was examined in the hydrogenation of toluene and the selective hydrogenation of CO_2_ to methane. A comparison between the activity and the selectivity of ruthenium catalysts supported on mesoporous supports was discussed. The results of the catalytic tests performed at several temperatures over metal supported on different mesoporous supports are presented in Figure 5 and Figure 6 (toluene hydrogenation) and Figure 7 and Figure 8 (carbon dioxide hydrogenation), including activity, selectivity, and structure–performance relationships. The supports (SBA-3, SBA-12, and SBA-15) did not show any activity in the hydrogenation reactions, while the reduced ruthenium catalysts were active in these processes. Methylcyclohexane, methane, and CO were the only reaction products obtained over the tested catalysts.

#### 2.6.1. Hydrogenation of Toluene

Gas-phase conversion of toluene (TL) to methylcyclohexane (MCH) was investigated at atmospheric pressure using 1 wt% Ru catalysts supported on SBA-3, SBA-12, and SBA-15 in a fixed-bed continuous-flow reactor. Prior to the reaction, the catalysts were reduced in situ under hydrogen at 450 °C for 2 h. Catalytic tests were subsequently carried out in the temperature range of 75–250 °C while maintaining a constant toluene concentration of 0.75 µmol/cm^3^. Among the investigated systems, the Ru/SBA-12 catalyst showed the highest hydrogenation performance, as evidenced by both a higher apparent reaction rate (r_t_; Figure 5 and Figure 6a) and an increased turnover frequency (TOF, s^−1^; Figure 6b), compared with the Ru/SBA-3 and Ru/SBA-15 catalysts.

For each catalyst, an increase in reaction temperature led to higher activity, with the highest values observed at 100 °C (Figure 5). At temperatures exceeding this optimum, a decline in catalytic performance was noted. According to literature reports, this reduction may result from dehydrogenation processes [58] and/or from cracking reactions of methylcyclohexane, the hydrogenation product of toluene [59]. In contrast, for ruthenium catalysts deposited on SBA supports, no cracking by-products were detected, and methylcyclohexane was the sole reaction product. This observation clearly suggests that the decrease in catalytic performance at elevated temperatures resulted from the reverse process, namely the dehydrogenation of MCH.

The catalytic performance in toluene hydrogenation is strongly influenced by the type of support, particularly its textural properties. The activity of the catalysts, expressed as the toluene hydrogenation rate, decreases with increasing dispersion of the active phase (Figure 6a), which—as demonstrated in the previous sections—is determined by the nature of the support. Consequently, the catalytic activity at 100 °C, expressed as the TOF, increases with increasing Ru particle size (Figure 6b).

The results presented in Figure 6a,b reveal distinct structure–activity relationships that can be rationalized in terms of ruthenium particle size and support pore architecture. Ru/SBA-15, with its ultra-small Ru nanoparticles (1.2 nm, 80% dispersion), exhibited the lowest activity in toluene hydrogenation, with turnover frequencies (TOF) of approximately ~0.11 s^−1^ at 100 °C. The confinement of Ru precursors within SBA-15’s narrow cylindrical mesopores (5–7 nm) during impregnation and subsequent reduction prevents particle aggregation but simultaneously restricts the growth of well-crystallized Ru nanoparticles. Ru/SBA-3, featuring intermediate-sized Ru particles (2.8 nm, 35% dispersion), demonstrated significantly enhanced activity with TOF values approaching ~0.32 s^−1^ at 100 °C. Ru/SBA-12 displayed the highest activity among the three catalysts, with TOF values reaching ~0.6 s^−1^ at 100 °C, despite having the lowest dispersion (23%) and largest Ru particle size (4.3 nm).

To verify whether further increasing the Ru particle size leads to additional activity enhancement, a reference Ru/SiO_2_ catalyst with an average Ru crystallite size of ~8 nm was also evaluated under identical conditions (Figure 6). Despite the presence of substantially larger metallic ensembles, this catalyst did not exhibit higher TOF values than Ru/SBA-12 (4.3 nm). In fact, its intrinsic activity was comparable, indicating that continuous particle growth beyond 4–5 nm does not further improve hydrogenation performance. This observation confirms the existence of an optimal particle size range for toluene hydrogenation. While increasing particle size from 1.2 nm to 4.3 nm significantly enhances intrinsic activity, further growth to ~8 nm does not provide additional benefits.

The three-dimensional pore structure of SBA-12, characterized by interconnected spherical cavities accessible through narrow windows, allows for the formation of larger, highly crystalline Ru nanoparticles within the cavity interiors. These well-faceted Ru crystallites exhibit superior intrinsic activity per surface site due to enhanced H_2_ dissociation on plane sites and stronger toluene adsorption, as evidenced by the XRD-visible Ru^0^ (101) reflection at 44° (2θ). This finding is consistent with the observations of Somorjai et al. [60], who investigated vapor-phase benzene and toluene hydrogenation over size-controlled Pt/SBA-15 catalysts (1.5–5.2 nm) and reported that the turnover frequency increases with Pt particle size, with optimal activity observed for Pt particles around 3.1 nm. This behavior was attributed to a favorable balance between corner/edge sites (active for H_2_ activation) and terrace sites (active for aromatic adsorption and hydrogenation). However, the substantially lower total ruthenium surface area in Ru/SBA-12 partially offsets the advantage of higher intrinsic activity, resulting in only a modest overall activity enhancement compared to Ru/SBA-3.

In summary, the experimental results for gas-phase toluene hydrogenation over Ru/SBA-3, Ru/SBA-12, and Ru/SBA-15 catalysts align well with established structure–activity principles for noble metal-catalyzed aromatic hydrogenation. The observed trend—Ru/SiO_2_ ≅ Ru/SBA-12 (largest particles, highest TOF per site) > Ru/SBA-3 (intermediate particles, balanced performance) > Ru/SBA-15 (smallest particles, lowest activity)—reflects the interplay between metal dispersion, particle crystallinity, and support pore topology.

The intrinsic activity differences observed among the Ru/SBA-3, Ru/SBA-12, and Ru/SBA-15 catalysts can be rationalized not only in terms of Ru particle size and crystallinity, but also by considering the accessibility of Ru sites imposed by the distinct pore architectures of the SBA supports. In the case of Ru/SBA-15, the ultra-small Ru nanoparticles (~1.2 nm) are highly dispersed and strongly confined within the long, cylindrical mesopores (5–7 nm). While this confinement effectively prevents particle growth and sintering, it can also limit the accessibility of Ru sites under reaction conditions due to diffusion constraints and steric shielding within the pore channels. As a result, not all Ru surface atoms quantified by CO chemisorption may actively participate in the catalytic reaction. Ru/SBA-3, with its smaller pore diameter (2–3 nm), exhibits intermediate Ru particle size (~2.8 nm) and moderate dispersion. The restricted pore dimensions likely hinder deep precursor penetration during impregnation, leading to a larger fraction of Ru species located on external surfaces or near pore entrances. This configuration improves the accessibility of Ru sites while maintaining sufficient metal dispersion, resulting in a balanced catalytic performance. In contrast, the 3D cage-like pore network of SBA-12 facilitates the formation of larger, more crystalline Ru nanoparticles (~4.3 nm), as evidenced by the appearance of XRD-detectable metallic Ru reflections. These particles are located within interconnected cavities that offer improved molecular accessibility compared to narrow, one-dimensional channels, while still providing geometric confinement. The larger, well-faceted Ru crystallites supply extended metallic ensembles and more exposed Ru^0^ facets, which are favorable for H_2_ dissociation and subsequent hydrogenation steps. Consequently, despite its lower Ru dispersion, Ru/SBA-12 exhibits the highest intrinsic activity (TOF), highlighting the combined role of particle size, crystallinity, and pore architecture in governing catalytic performance. These findings underscore the importance of rational catalyst design, where the selection of mesoporous support structure must be tailored to the desired balance between metal dispersion and nanoparticle crystallinity to optimize catalytic performance for specific reactions, such as toluene hydrogenation.

#### 2.6.2. Selective Hydrogenation of CO_2_ to Methane

The second reaction, in which the catalysts were tested, was the selective hydrogenation of carbon dioxide to methane. The catalytic performance of the three ruthenium catalysts in CO_2_ methanation (CO_2_ + 4H_2_ → CH_4_ + 2H_2_O) reveals significant differences in activity and methane selectivity as a function of reaction temperature (Figure 7). All three catalysts exhibit increasing CO_2_ conversion with rising temperature, approaching thermodynamic equilibrium conversion (>60%) at 450–500 °C, consistent with the exothermic nature of the Sabatier reaction (ΔH° = −165 kJ/mol). Ru/SBA-12 demonstrates the highest CO_2_ conversion across the entire temperature range (250–500 °C), achieving approximately 45.3% at 400 °C and 63.7% at 450 °C, followed by Ru/SBA-3 (34.0% at 400 °C, 52.9% at 450 °C), while Ru/SBA-15 exhibits the lowest conversion (27.6% at 400 °C, 48.5% at 450 °C). Similarly to the behavior observed in toluene hydrogenation, the Ru/SiO_2_ catalyst containing larger Ru crystallites (~8 nm) did not exhibit superior performance in CO_2_ methanation. Although this catalyst contains significantly larger Ru ensembles than Ru/SBA-12, no further improvement in CO_2_ conversion or TOF was observed. The activity (TOF) was comparable to that of Ru/SBA-12, indicating that the beneficial ensemble effect reaches a maximum within the 3–5 nm range. Methane selectivity for the Ru/SBA-12 catalyst remains above 80% over the entire investigated temperature range, whereas Ru/SBA-3 exhibits a maximum CH_4_ selectivity of 63% at 450 °C (Figure 7b).

Another important element of the response under study is the monitoring of carbon balance (CB). Carbon deposition theoretically does not occur if the H_2_/CO_2_ ratio is equal to or higher than the stoichiometric ratio [61]. Our studies were conducted at atmospheric pressure and at temperatures up to 500 °C. Temperatures above 500 °C can cause sintering of Ru particles and increase carbon deposition, leading to catalyst deactivation. Thus, temperature control is vital as the exothermic methanation reaction can result in an apparent temperature increase in large-scale operations [62]. For this reason, the CB was monitored and was in the range of 0.96–1.00 at each temperature, meaning that there was no degradation of carbon dioxide.

To further elucidate the role of the support in CO_2_ activation, CO_2_-TPD measurements were performed (Figure S3, Supplementary Information). All catalysts exhibit extremely low CO_2_ uptake and only very weak desorption features, consistent with the intrinsically low basicity of purely siliceous SBA materials. Importantly, no correlation is observed between the amount or strength of basic sites and catalytic performance. For example, Ru/SBA-15 shows slightly more discernible desorption features, yet it exhibits the lowest catalytic activity. These results indicate that support basicity does not govern the activity differences among the investigated catalysts. Instead, CO_2_ activation and subsequent methanation proceed predominantly on Ru metallic sites, and the observed activity trends are primarily determined by particle size effects and structure sensitivity rather than by variations in surface basicity.

The observed activity trend (Ru/SBA-12 > Ru/SBA-3 > Ru/SBA-15) inversely correlates with ruthenium dispersion (23% vs. 35% vs. 80%—Figure 8a) and particle size (4.3 nm vs. 2.8 nm vs. 1.2 nm—Figure 8b), providing strong evidence that the turnover frequency for CO_2_ methanation increases with increasing Ru particle size. This finding aligns with extensive literature demonstrating that CO_2_/CO methanation over Ru exhibits pronounced structure sensitivity, with turnover frequency (TOF) increasing with particle size due to the ensemble requirement for CO_2_ dissociation and CH_4_ formation. Truszkiewicz et al. [63] reported that CO methanation over Ru/carbon catalysts shows optimal activity for particles in the 3–5 nm range, with TOF decreasing sharply for particles below 2 nm due to insufficient ensemble sites for C-O bond cleavage and carbon hydrogenation. Panagiotopoulou et al. [64] observed similar behavior for CO methanation over Ru/TiO_2_, Al_2_O_3_, and MgO, with TOF increasing 2–3 fold as particle size increased from 2.1 to 4.5 nm, which they attributed to the requirement of larger Ru ensembles for efficient CO dissociative chemisorption. A similar relationship was demonstrated in our previous studies on ruthenium supported on silicalite-1 modified with solutions of alkali metal compounds [65]. The present results are fully consistent with these reports: Ru/SBA-3 (2.8 nm) and Ru/SBA-12 (4.3 nm) possess particle sizes close to the optimal 3–4 nm range, providing sufficient metallic ensembles for efficient CO_2_ activation and hydrogenation, whereas Ru/SBA-15’s ultra-small particles (1.2 nm) likely consist of Ru clusters with fewer than 10 atoms, which are sub-optimal for CO_2_ methanation despite offering the highest metal surface area. The inclusion of the Ru/SiO_2_ catalyst (~8 nm, Figure 8b) further strengthens this conclusion. While very small Ru clusters (<2 nm) are clearly sub-optimal for methanation due to insufficient ensemble size, excessively large crystallites do not provide additional intrinsic activity enhancement. Therefore, the present results clearly demonstrate the presence of an optimal Ru particle size range rather than a continuous increase in activity with crystallite size.

The quantitative relationships between methanation rate (Figure 8a) and methanation TOF (Figure 8b), and catalyst structure provide compelling evidence for structure sensitivity. The methanation rate at 450 °C exhibits a strong negative correlation with Ru dispersion (Pearson’s r = −0.988), decreasing from ~0.963 mmol_CO2_/(mmol_Ru_·s) for Ru/SBA-12 (23% dispersion) to ~0.879 for Ru/SBA-3 (35% dispersion) and ~0.725 mmol_CO2_/(mmol_Ru_·s) for Ru/SBA-15 (80% dispersion). Conversely, the turnover frequency (TOF) shows a strong positive correlation with particle size (Pearson’s r = 0.959), increasing from ~0.954 mmol_CO2_/(mmol_Ru_surface_·s) for Ru/SBA-15 (1.2 nm) to ~2.278 for Ru/SBA-3 (2.8 nm) and reaching ~4.185 mmol_CO2_/(mmol_Ru_surface_·s) for Ru/SBA-12 (4.3 nm). These trends unequivocally demonstrate that larger Ru particles exhibit higher intrinsic activity per surface metal atom in CO_2_ methanation. This means that higher activity is shown by ruthenium centers located on large metal crystallites. However, the fact that there are far fewer of them (due to low dispersion) translates into lower overall activity. Similar correlations, turnover frequency of CO_2_ conversion on the size of crystallites, were shown by the authors of a paper [66] studying the effect of Ni crystallite size on the activity of CO_2_ methanation, as well as the authors of the paper [67] studying Ru/MnO_x_ catalysts.

A comparison of the CO_2_ methanation rate value obtained for the best 1 wt% Ru/SBA-12 catalyst (426 mmol_CO2_/(g_cat_·h) at 300 °C) with results reported in the literature shows that our material is a highly promising catalyst. For instance, ruthenium supported on Al_2_O_3_ (10 wt% Ru) or MgO (10 wt% Ru) achieved rates of 350 and 320 mmol_CO2_/(g_cat_·h) at 240 °C [68], respectively, while a 1Ru/Sil-0.1 NaOH (1 wt% Ru) catalyst reached 536 mmol_CO2_/(g_cat_·h) at 250 °C [65]. In comparison with other oxide-supported systems, Panagiotopoulou et al. reported reaction rates for 5 wt% Ru/Al_2_O_3_ and 5 wt% Ru/TiO_2_ at 330 °C of 110.7 and 64.8 μmol_CO2_/(g_cat_·s), respectively [64]. Furthermore, Dreyer et al. demonstrated that ruthenium catalysts synthesized via flame spray pyrolysis exhibit significant support-dependent activity, with turnover frequency (TOF) values at 300 °C for 5 wt% Ru on Al_2_O_3_ and CeO_2_ reaching 0.38 s^−1^ and 0.15 s^−1^, respectively [69]. In contrast, ruthenium supported on carbon nanofibers (0.5 wt% Ru/CNFs) showed much lower activity, with CO_2_ conversion only initiating at 300 °C and reaching 50% at temperatures as high as 500 °C [70]. These comparisons underscore that the 1 wt% Ru/SBA-12 catalyst, characterized by an optimal particle size of 4.3 nm and high TOF (4.185 s^−1^ at 450 °C), provides competitive or superior activity to systems with much higher metal loadings.

In summary, the superior performance of Ru/SBA-12 and Ru/SBA-3 compared to Ru/SBA-15 underscores an important design principle for CO_2_ methanation catalysts: achieving an optimal particle size window (approximately 3–5 nm) is more critical than either maximizing dispersion or promoting unrestricted particle growth beyond this range. While Ru/SBA-15’s high dispersion (80%) offers maximum metal surface area, its ultra-small particles (1.2 nm) suboptimal for CO_2_ methanation. This finding has significant implications for catalyst design—supports that stabilize particles in the 3–5 nm range (like SBA-3’s 2–3 nm pores or SBA-12’s cage structures) are preferred over those enforcing sub-2 nm particles through strong confinement effects. The present results confirm that silica-supported Ru catalysts, despite lacking reducible oxide supports that can enhance CO_2_ activation via oxygen vacancies, remain highly active when particle size is optimized in the 3–4 nm range. The mesoporous SBA supports provide additional advantages beyond simple SiO_2_: (1) SBA-3’s small pores naturally template Ru particles to the optimal size range during impregnation, (2) SBA-12’s 3D cage structure facilitates H_2_ and CO_2_ diffusion while preventing excessive sintering within cages, and (3) SBA-15’s large channels, while producing sub-optimal particle size for methanation, enable superior mass transport of reactants and products, which may become advantageous at higher conversions where internal diffusion limitations arise. These structure–activity–support correlations provide a rational framework for selecting mesoporous supports based on the target reaction: for CO_2_ methanation, supports that enforce intermediate particle sizes (3–4 nm) via pore confinement effects (SBA-3, SBA-12) outperform those stabilizing ultra-small clusters (SBA-15).

## 3. Materials and Methods

### 3.1. Preparation of Supports

All supports were produced via the hydrothermal method in acidic conditions.

SBA-3 support was synthesized following a method analogous to that previously described in Ref. [25]. Briefly, 8.3 g of cetyltrimethylammonium bromide (Aldrich, Saint Louis, MO, USA) was dissolved in 165 cm^3^ of deionized water. Subsequently, 23 cm^3^ of tetraethyl orthosilicate (Aldrich) was slowly introduced dropwise. The mixture was stirred for 15 min before adding 34 cm^3^ of 38% HCl (Polish Chemicals Reagents, Gliwice, Poland). After 30 min of additional stirring, a second portion of 36 cm^3^ of hydrochloric acid was added, leading to the formation of a white precipitate. The suspension was maintained under stirring at room temperature for 2 h and then left to age under static conditions at room temperature for 8 days. The solid product was recovered by filtration, thoroughly washed, dried in air at room temperature.

SBA-12 was synthesized using a procedure adapted from that described by Kumar et al. [46]. In a typical synthesis, 4 g of Brij-76 (Aldrich) was dissolved in a solution containing 80 cm^3^ of 2 M HCl and 20 cm^3^ of distilled water, followed by stirring at 40 °C for 2 h. Tetraethyl orthosilicate (9.43 cm^3^) was then slowly added dropwise over a period of 30 min. The mixture was maintained under stirring at 40 °C for 20 h and subsequently aged in an oven at 95 °C for 24 h. After cooling, the solid product was collected by filtration, washed with distilled water, and dried overnight at room temperature.

SBA-15 was obtained following a synthesis procedure based on the method reported in Ref. [71]. In this procedure, 4.0 g of Pluronic P123 (Aldrich) was dissolved in a mixture of 30 cm^3^ of deionized water and 116 cm^3^ of 2 M HCl (Polish Chemicals Reagents, Gliwice, Poland). The solution was stirred at 35 °C until complete homogenization was achieved. Then, 9.1 cm^3^ of tetraethyl orthosilicate (Aldrich) was introduced. The resulting mixture was kept under stirring at 35 °C for 20 h and subsequently subjected to aging at 90 °C for 24 h. The formed white solid was recovered by filtration, thoroughly washed with distilled water, and air-dried at room temperature.

For all three supports, the final step involved calcination in air at 550 °C for 8 h to remove the organic template.

### 3.2. Preparation of Catalysts

Ruthenium-based catalysts were synthesized using the incipient wetness impregnation technique, with an aqueous solution of RuCl_3_·nH_2_O (Aldrich) as the metal source. The ruthenium loading in all samples was fixed at 1 wt%. An appropriate amount of the support was introduced to the RuCl_3_·nH_2_O solution, followed by solvent evaporation. The resulting materials were subsequently dried at 105 °C for 24 h and denoted as Ru/SBA-3-D, Ru/SBA-15-D, and Ru/SBA-12-D.

### 3.3. Activation of Catalysts

Before CO chemisorption experiments and prior to characterization by inductively coupled plasma optical emission spectroscopy (ICP-OES), X-ray diffraction (XRD), Transmission electron microscopy (TEM) and low-temperature nitrogen adsorption–desorption (BET), the dried materials-Ru/SBA-3-D, Ru/SBA-12-D, and Ru/SBA-15-D were subjected to hydrogen reduction in a fixed-bed flow reactor. Initially, the system was purged at room temperature with helium (99.99%, Linde, Pullach im Isartal, Germany) at a flow rate of 150 cm^3^/min, after which the gas was switched to hydrogen at the same flow rate. Following a 15 min stabilization period, the hydrogen flow was decreased to 50 cm^3^/min and the temperature was increased at a rate of 10 °C/min. Once the reduction temperature of 450 °C was reached, the samples were maintained under hydrogen for 2 h. Subsequently, the catalysts were cooled to room temperature under hydrogen flow and finally purged with helium for 15 min. The reduced materials were designated as Ru/SBA-3, Ru/SBA-15, and Ru/SBA-12.

### 3.4. Catalysts Characterization

#### 3.4.1. Determination of Surface Area, Pore Volume and Pore Diameter

Structural properties of the catalysts were characterized by nitrogen adsorption–desorption measurements performed at −196 °C using a Micromeritics ASAP 2010 sorptometer (Micromeritics, Norcross, GA, USA). Before the analysis, the samples were degassed at 300 °C for 4 h to remove adsorbed moisture and gases.

The specific surface area was calculated according to the Brunauer–Emmett–Teller (BET) method, and the total pore volume was obtained from the amount of nitrogen adsorbed at a relative pressure (p/p_0_) of 0.99. The average pore diameter was derived using the Barrett–Joyner–Halenda (BJH) model applied to the desorption branch of the isotherm, while the micropore volume was estimated by the t-plot method.

#### 3.4.2. Determination of Ruthenium Content

The amount of ruthenium in the catalysts reduced at 450 °C for 2 h was quantified by inductively coupled plasma optical emission spectroscopy (ICP-OES) using a Varian Vista-MPX instrument (Varian, Inc. (currently part of Agilent Technologies), Palo Alto, CA, USA).

Elemental analysis revealed that all samples contained nearly identical Ru loadings, amounting to 0.96, 0.98, and 0.96 wt% for the SBA-3, SBA-15, and SBA-12 supported catalysts, respectively.

#### 3.4.3. Temperature-Programmed Reduction with Hydrogen—H_2_-TPR

To investigate the reduction behavior of the catalysts, we performed temperature-programmed reduction measurements using a Pulse ChemiSorb 2705 (Micromeritics, Norcross, GA, USA) instrument.

For each experiment, approximately 50 mg of dried materials—Ru/SBA-3-D, Ru/SBA-12-D, and Ru/SBA-15-D—was subjected to a 10 vol% H_2_–Ar flow (99.999%, Linde) at 30 cm^3^/min. The reduction was carried out over a temperature range of 50–550 °C with a linear heating rate of 10 °C/min. To ensure a direct comparison, all H_2_-TPR profiles presented in this study were normalized to a sample mass of 100 mg.

#### 3.4.4. Temperature-Programmed Desorption of CO_2_—CO_2_-TPD

Surface basicity was determined through CO_2_ temperature-programmed desorption (CO_2_-TPD) using a Micromeritics PulseChemiSorb 2705 (Micromeritics, Norcross, GA, USA) equipped with a thermal conductivity detector. For each measurement, approximately 150 mg of Ru catalyst was activated at 400 °C for 30 min under helium flow. Following cooling to 50 °C, the samples were exposed to CO_2_ for 30 min. A subsequent 60 min helium purge removed physisorbed species. Desorption was recorded while heating the catalysts from 50 °C to 350 °C at 10 °C/min, concluding with a 30 min isothermal hold at the maximum temperature. The resulting profiles were normalized to 1 g of sample mass.

#### 3.4.5. X-Ray Diffraction Analysis (XRD)

The crystalline structure of the materials was examined by X-ray powder diffraction using a Bruker AXS D8 Advance diffractometer (Bruker, Billerica, MA, USA). Ni-filtered CuKα radiation (λ = 1.54056 Å) was employed. Diffraction patterns were recorded over two 2θ ranges: a low-angle range from 0.6° to 8° and a wide-angle range from 35° to 80°.

#### 3.4.6. Transmission Electron Microscopy (TEM)

Transmission electron microscopy (TEM) images were recorded on a JEOL 2000 microscope (Jeol, Tokyo, Japan) operating at accelerating voltage of 80 kV.

#### 3.4.7. Determination of Ru Dispersion—CO Chemisorption

Carbon monoxide chemisorption measurements were carried out using the static method at 100 °C on a Micromeritics ASAP 2010C sorptometer (Micromeritics, Norcross, GA, USA).

Before the analysis, the freshly dried materials (Ru/SBA-3-D, Ru/SBA-12-D, and Ru/SBA-15-D) were reduced in hydrogen at 450 °C for 2 h (as described in the Section 3.3), followed by in situ pretreatment to remove adsorbed gases from the surface. The pretreatment procedure involved evacuation at room temperature for 15 min, heating at 360 °C under vacuum for 60 min, subsequent reduction in hydrogen flow (40 cm^3^/min, 99.999%, Linde, Pullach im Isartal, Germany) at 360 °C for 60 min, and a final evacuation at 360 °C for 120 min. Ruthenium dispersion was determined based on the amount of adsorbed CO. Using the volume of carbon monoxide corresponding to monolayer coverage (ν_m_), the metallic surface area (S, in m^2^/g_Ru_) was calculated according to Equation (1) [72]:

(1) $$S=\frac{v_{m}\cdot N_{A}\cdot n\cdot a_{m}\cdot 100}{22414\cdot m\cdot \mathrm{wt}}$$

where *ν_m_* is the volume of adsorbed CO (cm^3^), *N_A_* is Avogadro’s number (6.022 × 1023 mol^−1^), *n* is the chemisorption stoichiometry (*n* = 1 for CO), *a_m_* is area occupied by 1 Ru atom (8.17 × 10^20^ m^2^/atom), *m* is the sample mass (g), and *wt%* represents the metal loading.

The dispersion (*D*) of the active phase was calculated according to Equation (2):

(2) $$D=\frac{S\cdot M}{a_{m}\cdot N_{A}}$$

where *S* represents the metallic surface area, *M* is the atomic weight of ruthenium (101 g/mol), *N_A_* is Avogadro’s number, and *a_m_* is the surface area occupied by one ruthenium atom (8.17 × 10^20^ m^2^/atom).

Assuming a spherical shape of metal particles, the mean crystallite size of ruthenium was calculated using Equation (3):

(3) $$d=\frac{6000}{\rho \cdot S}$$

where *d* represents crystallite size in nm, ρ—density of ruthenium (12.37 g/cm^3^) and *S*—metal surface area [m^2^/g_Ru_].

### 3.5. Catalytic Tests

#### 3.5.1. Hydrogenation of Toluene to Methylcyclohexane

Toluene hydrogenation was carried out at atmospheric pressure in a fixed-bed flow reactor using hydrogen as the carrier gas. Approximately 25 mg of freshly dried catalyst was loaded into the reactor and reduced in situ with hydrogen (100 cm^3^/min, 99.99%, Linde) at 450 °C for 2 h. After reduction, the reactor temperature was adjusted to the desired reaction temperature, and a hydrogen stream (50 cm^3^/min) passed through a toluene saturator equilibrated at 10 °C (99.8%, Aldrich) was introduced. The reactor had an inner diameter of 8.0 mm, and the catalyst bed volume ranged from 0.15 to 0.25 cm^3^ depending on the catalyst density. The toluene concentration in the hydrogen feed was maintained at 0.75 μmol/cm^3^. The catalysts were heated at 10 °C/min until reaching the target reaction temperature (50–200 °C). Reaction products were analyzed using a gas chromatograph equipped with a RESTEK-MXT-1 capillary column (Restek GmbH, Bad Homburg, Germany). Catalytic activity was expressed as the apparent rate (r), calculated according to Equation (4):

(4) $$r_{t}=\frac{F\cdot Y\cdot C}{N}$$

where *F* is the total feed flow rate (cm^3^/min), *Y* is the fractional conversion, *C* is the toluene concentration in the feed (mol_Tl_/cm^3^), and *N* is the ruthenium content (mol_Ru_) in the sample. Turnover frequency (TOF, s^−1^) was determined by dividing the number of molecules converted per second by the number of active ruthenium atoms measured via CO chemisorption.

#### 3.5.2. Hydrogenation of CO_2_

Prior to CO_2_ methanation, the catalyst samples were reduced in hydrogen at 450 °C for 120 min. Activity tests were conducted using a feed composed of CO_2_ and H_2_ in a stoichiometric ratio of 1:4, diluted with helium to achieve a 1:1 ratio of reactants to He. The resulting feed composition was CO_2_:H_2_:He = 1:4:5, with a total flow rate of 100 cm^3^/min (all gases 99.999%, Linde, Pullach im Isartal, Germany). In each experiment, 50 mg of catalyst was loaded into a fixed-bed reactor operating at atmospheric pressure over the temperature range of 250–500 °C. At each temperature, the reaction was carried out for 30 min to ensure steady-state conditions. Gaseous reactants and products were analyzed using an SRI Multiple Gas Analyzer #1 GC (SRI Instruments, Torrance, CA, USA). This system was equipped with a silica gel packed column, a Molecular Sieve 13X, and a TCD detector, operating with helium as the carrier gas.

CO_2_ conversion (*X_CO_*_2_) was calculated according to Equation (5):

(5) $$X_{{CO}_{2}}=\frac{C_{CO,out}+C_{{CH}_{4},out}}{C_{{CO}_{2},out}+C_{CO,out}+C_{{CH}_{4},out}}\times 100$$

where *C_CO,out_*, *C_CO_*_2*,out*_, and *C_CH_*_4_*_,out_* represent the outlet concentrations of CO, CO_2_, and CH_4_, respectively.

Selectivities toward CH_4_ (*S_CH_*_4_) and CO (**S_CO_**) were determined using Equations (6) and (7) based on the outlet concentrations of CH_4_ (*C_CH_*_4*,out*_) and CO (*C_CO,out_*).

(6) $$S_{{CH}_{4}}=\frac{C_{{CH}_{4},out}}{C_{{CH}_{4},out}+C_{CO,out}}\times 100$$

(7) $$S_{CO}=\frac{C_{CO,out}}{C_{{CH}_{4},out}+C_{CO,out}}\times 100$$

Finally, the carbon balance (*CB*) was verified using Equation (8) considering the outlet concentrations of CH_4_ (*C_CH_*_4*,out*_), CO_2_ (*C_CO_*_2*,out*_), and CO (*C_CO,out_*), as well as the inlet CO_2_ concentration (*C_CO_*_2*,in*_).

(8) $$Carbon\;balance\;(CB)=\frac{C_{{CH}_{4},out}+C_{{CO}_{2},out}+C_{CO,out}}{C_{{CO}_{2},in}}$$

## 4. Conclusions

This comprehensive investigation of 1 wt% ruthenium catalysts supported on three distinct mesoporous silica architectures—SBA-15, SBA-12, and SBA-3—has elucidated fundamental structure–activity relationships governing catalytic performance in CO_2_ methanation and gas-phase toluene hydrogenation, demonstrating that mesoporous support topology exerts decisive control over ruthenium nanoparticle size, dispersion, crystallinity, and reducibility, with profound consequences for reactions exhibiting contrasting degrees of structure sensitivity.

The catalytic performance of the investigated 1 wt% Ru catalysts in both studied hydrogenation reactions is governed by the same particle-size trend, yet the degree of structure sensitivity differs markedly. In toluene hydrogenation, activity decreases in the order Ru/SBA-12 > Ru/SBA-3 > Ru/SBA-15, i.e., inversely to Ru dispersion and proportionally to particle size. The largest, well-crystallized Ru particles (4.3 nm) in the cage-like SBA-12 structure exhibit the highest turnover frequency (TOF ≈ 0.6 s^−1^ at 100 °C), while the ultra-small clusters (1.2 nm) confined in SBA-15 channels show the lowest intrinsic activity. In CO_2_ methanation, the same sequence is observed, but the differences are significantly more pronounced: Ru/SBA-12 >> Ru/SBA-3 > Ru/SBA-15. The TOF at 450 °C increases from ~0.95 s^−1^ for the 1.2 nm particles in Ru/SBA-15 to ~4.2 s^−1^ for the 4.3 nm particles in Ru/SBA-12. Ultra-small Ru clusters (<2 nm) lack sufficient large metallic ensembles required for efficient CO_2_ dissociation and subsequent methane formation, leading to dramatically reduced performance despite the highest metal surface area.

These results demonstrate that the pore architecture of the mesoporous silica support determines the achievable Ru particle size, which in turn controls catalytic behavior. For toluene hydrogenation, high dispersion remains beneficial, whereas for CO_2_ methanation, optimal performance is only achieved with Ru particles in the 3–4 nm range. Consequently, cage-like SBA-12 and intermediate-pore SBA-3 are superior supports for CO_2_ methanation, while strong confinement in SBA-15, although excellent for maximizing dispersion, is counterproductive for this particular reaction.

## Figures and Tables

**Table 1:** Characterization of supports calcined at 550 °C and ruthenium catalysts reduced at 450 °C.

Sample Code	Physical Characterization of Supports and Catalysts							
	Method of Activation	BET Surface Area ^a^, m^2^/g	S_micro_^b^ m^2^/g	S_ext_^c^ m^2^/g	Total Pore Volume ^d^, cm^3^/g	V_micro_^e^ cm^3^/g	V_meso_^f^ cm^3^/g	Average Pore Diameter ^g^, nm
SBA-3	Calcination (air, 8 h, 550 °C)	1032	968	64	0.51	0.45	0.06	2.2
SBA-12		845	6	839	0.82	<0.01	0.81	3.4
SBA-15		754	130	624	0.90	0.05	0.85	5.8
Ru/SBA-3	Reduction (hydrogen, 2 h, 450 °C)	995	937	58	0.48	0.43	0.05	2.3
Ru/SBA-12		787	4	783	0.76	<0.01	0.75	3.4
Ru/SBA-15		712	111	601	0.85	0.04	0.81	5.8

^a^ BET specific surface area, ^b^ surface of micropores, ^c^ external surface, ^d^ total pore volume, ^e^ micropore volume, ^f^ mesopore volume, ^g^ BJH desorption average pore diameter.

**Table 2:** CO chemisorption data for ruthenium catalysts reduced at 450 °C, 2 h.

Catalyst	Method of Activation	CO Volume Adsorbed, cm^3^/g	Ru Dispersion, %	Ru Surface Area m^2^/g_Ru_	Mean Size of Ru, nm
Ru/SBA-3	Reduction (H_2_, 2 h, 450 °C)	0.77	35	170	2.8
Ru/SBA-12		0.51	23	113	4.3
Ru/SBA-15		1.77	80	388	1.2
Ru/SiO_2_		0.28	13	61	7.9

## Data Availability

The data presented in this study are available on request from the corresponding author.

## Supplementary Materials

The following supporting information can be downloaded at: https://www.mdpi.com/article/10.3390/molecules31071130/s1; Table S1: Characterization of SiO_2_ calcined at 550 °C and Ru/SiO_2_ catalysts reduced at 450 °C; Figure S1: N_2_ adsorption/desorption isotherms (a) and pore size distribution (b) for Ru/SBA samples; Figure S2: TEM micrographs of the Ru/Sil-12 catalyst; Figure S3: CO_2_-TPD profiles for Ru/SBA catalysts pre-reduced in H_2_ at 450 °C.
