# Supplementary material for: Catalytic Synergy: Mesoporous Silica and Ruthenium—Structure–Activity Relationships in CO2 Methanation and Toluene Hydrogenation
Source: Molecules. 2026 Mar 29;31(7):1130. doi: 10.3390/molecules31071130 (PMC13075191; doi:10.3390/molecules31071130)
Supplement: Supplementary file 1 [file molecules-31-01130-s001.zip › molecules-4146202-supplementary.pdf]

Catalytic Synergy: Mesoporous Silica and Ruthenium—Structure–Activity Relationships in CO_2 Methanation and Toluene Hydrogenation

Ewa Janiszewska, Mariusz Pietrowski, Michał Zieliński*

Faculty of Chemistry, Adam Mickiewicz University, Uniwersytetu Poznańskiego 8, 61-614 Poznań, Poland; eszym@amu.edu.pl (E.J.), mariop@amu.edu.pl (M.P.)
* Correspondence: mardok@amu.edu.pl (M.Z.),

Table S1. Characterization of SiO_2 calcined at 550 °C and Ru/SiO_2 catalysts reduced at 450 °C.

Sample code	Method of activation	BET surface area, m^2/g	Total pore volume, cm^3/g	Average pore diameter, nm
SiO_2	calcination (air, 8 h, 550 °C)	396	0.68	6.9
Ru/SiO_2	reduction (hydrogen, 2 h, 450 °C)	366	0.63	6.8

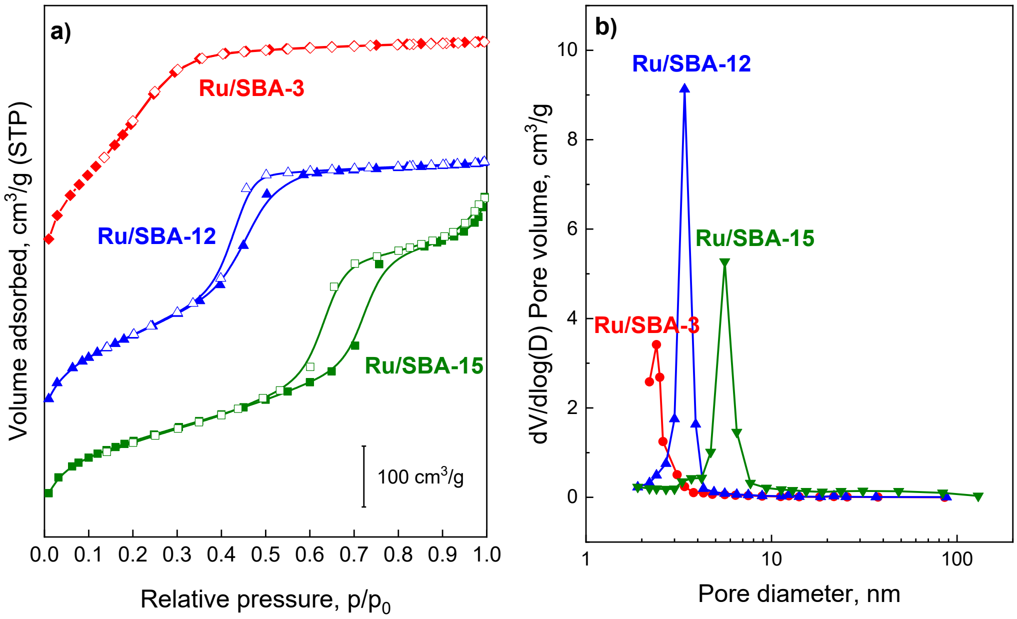

Figure S1. N_2 adsorption/desorption isotherms (a) and pore size distribution (b) for Ru/SBA samples.

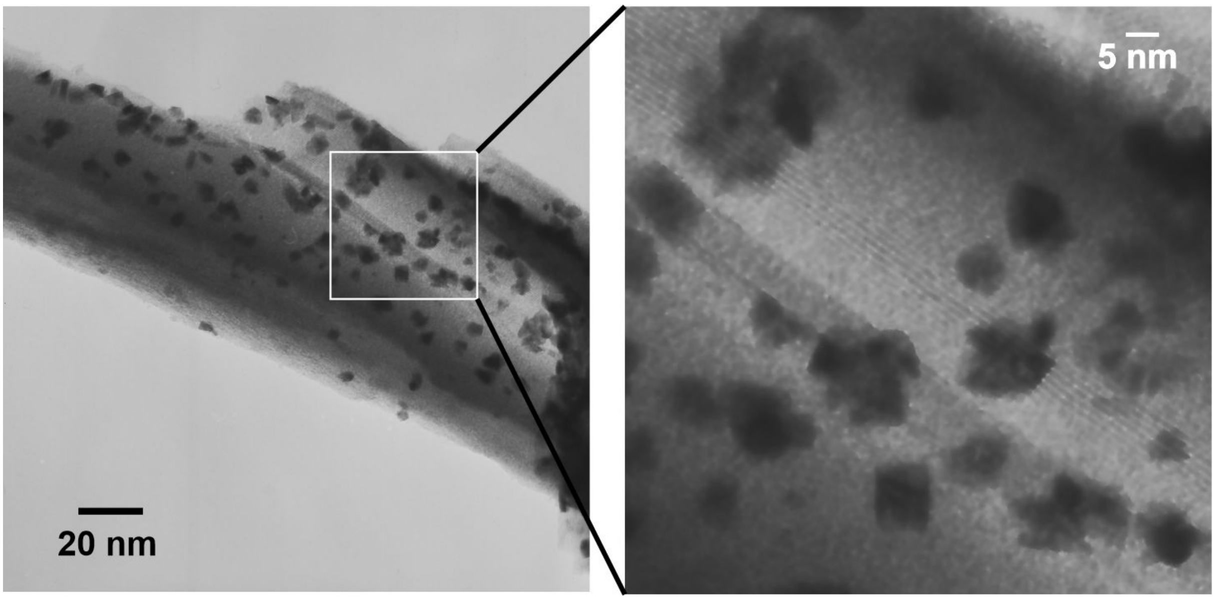

Figure S2. TEM micrographs of the Ru/Sil-12 catalyst.

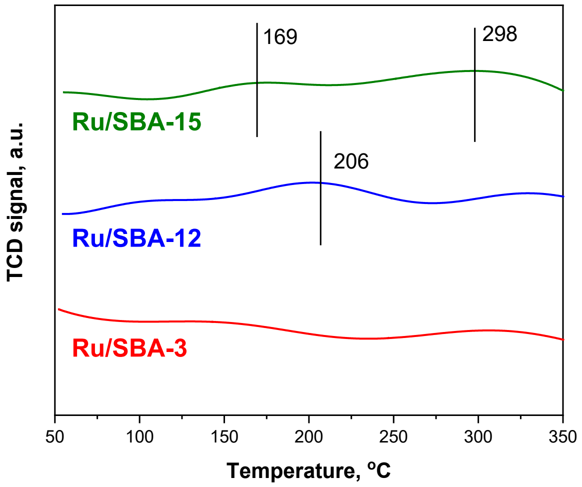

Figure S3. CO_2-TPD profiles for Ru/SBA catalysts pre-reduced in H_2 at 450°C.
CO_2-TPD condition: saturation at 50 °C under CO_2 flow for 60 min; heating at 10 °C/min under pure He flow.
